# Pesticidal and pest repellency activities of rhizomes of *Drynaria quercifolia* (J. Smith) against *Tribolium castaneum* (Herbst)

**DOI:** 10.1186/0717-6287-47-51

**Published:** 2014-10-01

**Authors:** Alam Khan, Md Hedayetul Islam, Md Ekramul Islam, Md Abdul Alim Al-Bari, Mst Shahnaj Parvin, Mohammed Abu Sayeed, Md Nurul Islam, Md Ekramul Haque

**Affiliations:** Department of Pharmacy, Rajshahi University, Rajshahi, Bangladesh; Institute of Food and Radiation Biology, AERE, Savar, Dhaka, 1349 Bangladesh; Department of Pharmacy, International Islamic University of Chittagong, Chittagong, Bangladesh; Department of zoology, Rajshahi University, Rajshahi, Bangladesh

**Keywords:** *Drynaria quercifolia*, Ethanol extract, Methanol soluble fraction, 3,4-dihydroxybenzoic acid, *Tribolium castaneum*

## Abstract

**Background:**

*Tribolium castaneum* (Herbst) is a harmful pest of stored grain and flour-based products in tropical and subtropical region. In the present study, rhizome of *Drynaria quercifolia* (J. Smith) was evaluated for pesticidal and pest repellency activities against *T. castaneum*, using surface film method and filter paper disc method, respectively. In addition, activity of the isolated compound 3,4-dihydroxybenzoic acid was evaluated against the pest.

**Results:**

Chloroform soluble fraction of ethanol extract of rhizome of *D. quercifolia* showed significant pesticidal activity at doses 0.88 to 1.77 mg/cm^2^ and significant pest repellency activity at doses 0.94 to 0.23 mg/cm^2^. No pesticidal and pest repellency activity was found for petroleum ether, ethyl acetate and methanol soluble fractions of ethanol extract as well as for 3,4-dihydroxybenzoic acid.

**Conclusion:**

Considering our findings it can be concluded that chloroform soluble fraction of rhizome of *D. quercifolia* is useful in controlling *T. castaneum* of stored grain and flour-based products.

**Electronic supplementary material:**

The online version of this article (doi:10.1186/0717-6287-47-51) contains supplementary material, which is available to authorized users.

## Background

Pests/insects often cause extensive damage to stored grain products, which is a serious problem throughout the world [1]. Certain pests can exist under a wide range of conditions and can attack products at all phases of storage and distribution. More than 2000 species of storage pests annually destroy approximately one third of the world’s food products. In many areas of the world, locally available materials are used to protect stored products against damage caused by pest infestation. Although synthetic pesticides are commonly used to control pests, is now causing concern because of environmental hazards, pests resistance and toxicity to mammals. Pesticides of plant origin, because of their high degree of tolerance by the mammals, are particularly desired for application against pests of fodders, fruits, vegetables and stored grains [2, 3]. The using of plant extracts in pest control has been practiced for at least two millennia, when botanical pesticides were considered important products for pest management in Ancient China, Egypt, India, Greece [4, 5]. Jacobson (1989) and Ketkar et al. (1976) have reviewed the effectiveness of plant derivatives for use against grain pests [6, 7]. In spite of the wide-spread recognition that many plants possess pesticidal properties, only a small number of pest control products directly obtained from plants [8, 9].

*Tribolium castaneum* (Herbst) is a major pest of stored flour and flour-based products in all tropical and subtropical countries of the world. Their presence in a stored food results in contamination and substantial economic damage due to loss of the products and a decrease in nutritional value. It is resistant to almost all organophosphorus pesticides. Dyte and Blackman (1972) reported that almost all of the strains of *T. castaneum* have become resistant to malathion [10]. The occurrence of malathion resistance by different strains of *T. castaneum* has given an extra impetus to search for alternative way for the control of this pest. *Drynaria quercifolia* J. Smith (syn. *Polypodium quercifolium*, Fam. Polypodiaceae), locally known as Gurar, is a parasitic fern [11, 12] that is widely distributed in Bangladesh, India and Thailand [12, 13]. The present study was aimed to determine the pesticidal and pest repellency activities of rhizomes of *D. quercifolia* against *T. castaneum*. Moreover, an antibacterial compound 3,4-dihydroxybenzoic acid was isolated from the rhizome of the plant and its activity against *T. castaneum* was also evaluated.

## Results

### Pesticidal activity

**Figure 1:**
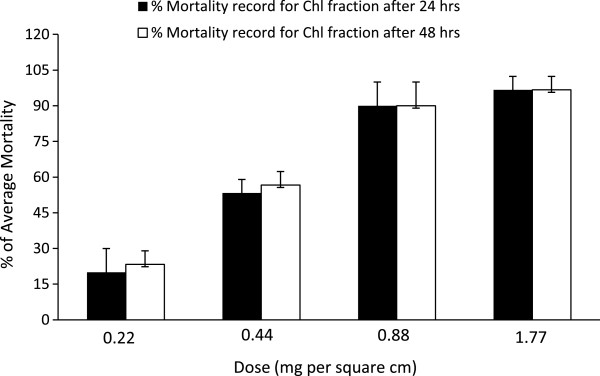
**Mortality records of chloroform fraction after 24 and 48 h.** Average mortality records were increased with dose of chloroform (Chl) fraction. On increasing the duration of chloroform fraction exposure to the pest, the mortality records were not significantly varied. Standard deviations are shown as error bar at the top of each column.

In our experiment, at 24 h duration of exposure, chloroform soluble fraction of the plant was observed for significant pesticidal activity against *T. castaneum* (Table 1). With the increment of doses the mortality record was up-regulated i.e. highest mortality record (96.60%) was observed for dose 1.77 mg/cm^2^ and lowest mortality record (20.00%) was observed for the dose 0.22 mg/cm^2^ (Table 1, 2, Figure 1). LD_50_ of chloroform fraction for 24 h duration of exposure was 0.40 mg/cm^2^ (Table 2). When duration of exposure was increased (48 h), mortality record was little increased and LD_50_ little reduced 0.37 mg/cm^2^ (Table 2). No pesticidal activity was found for petroleum ether, ethyl acetate and methanol soluble fractions of the plant. The compound 3,4-dihydroxybenzoic acid that was isolated from ethyl acetate fraction (also detected in chloroform fraction) did not showed pesticidal activity against *T. castaneum*.

**Table 1:** **Observation of screening for pesticidal activity for chloroform ext. (by surface film test) after 24 hours and 48 hours**

Dose (mg/cm ^2^)	#	Mortality record for applied pests			
		Record after 24 hours	Average ± SD record after 24 hours	Record after 48 hours	Average ± SD record after 48 hours
1.77	10	10	9.66 ± 0.57	10	9.66 ± 0.57
1.77	10	10		10	
1.77	10	9		9	
0.88	10	10	9.00 ± 1.00	10	9.00 ± 1.00
0.88	10	9		9	
0.88	10	8		8	
0.44	10	5	5.33 ± 0.57	6	5.66 ± 0.57
0.44	10	6		6	
0.44	10	5		5	
0.22	10	2	2.00 ± 1.00	2	2.33 ± 0.57
0.22	10	1		2	
0.22	10	3		3	
Control	10	0	0.00	0	0.00

# = Number of pests applied per petridish.

**Table 2:** **LD**_**50**_ **calculation for the pesticidal activity using probit analysis**

Recording time	Dose (mg/cm ^2^)	% of Average mortality	% Corrected mortality	Regression equation	LD _50_ (mg/cm ^2^)	95% Confidence limits	
						Lower	Upper
Record after 24 hours	1.77	96.66	97	Esimate 1	0.40	7.99	6.41
	0.88	90.00	90	Y = 1.72 + 3.10X			
	0.44	53.33	53	Esimate 2			
	0.22	20.00	20	Y = 1.61 + 3.19X			
Record after 48 hours	1.77	96.66	97	Esimate 1	0.37	7.33	15.65
	0.88	90.00	90	Y = 1.93 + 2.97X			
	0.44	56.66	57	Esimate 2			
	0.22	23.33	23	Y = 1.88 + 3.02X

### Pest repellency activity

In pest repellency study, chloroform fraction showed good repellency property at all tested doses (0.94 to 0.23 mg/cm^2^) (Table 3, Figure 2). Observations of first hours not significantly differ from the observations of subsequent hours (second, third, fourth and fifth hours). Like pesticidal activity, pest repellency activity also increased with doses. Petroleum ether, ethyl acetate and methanol soluble fractions as well as 3,4-dihydroxybenzoic acid were also subjected to pest repellency test, but no activity was found.

**Table 3:** **Pest repellency records and percent repulsions (PR) for chloroform soluble fraction of rhizome of** ***D. quercifolia*** **J. Smith**

Dose (mg/cm ^2^)	#	Repellency record														
		Hourly observation					Average of hourly observation (Nc)					Percent repulsion (PR) PR = (Nc - 5) х 20%				
		1 h	2 h	3 h	4 h	5 h	1 h	2 h	3 h	4 h	5 h	1 h	2 h	3 h	4 h	5 h
0.94	10	9	10	10	10	10	9.6	9.6	9.6	10.0	10.0	93.2%	93.2%	93.2%	100%	100%
0.94	10	10	10	10	10	10										
0.94	10	10	9	9	10	10										
0.47	10	9	10	10	10	10	9.0	9.3	9.3	9.6	9.6	80.0%	86.6%	86.6%	93.2%	93.2%
0.47	10	10	8	8	9	10										
0.47	10	8	10	10	10	9										
0.23	10	6	8	8	8	8	8.0	8.6	8.3	9.3	9.3	60.0%	73.2%	66.6%	86.6%	86.6%
0.23	10	10	9	9	10	10										
0.23	10	8	9	8	10	10										

# = Number of pests applied.

**Figure 2:**
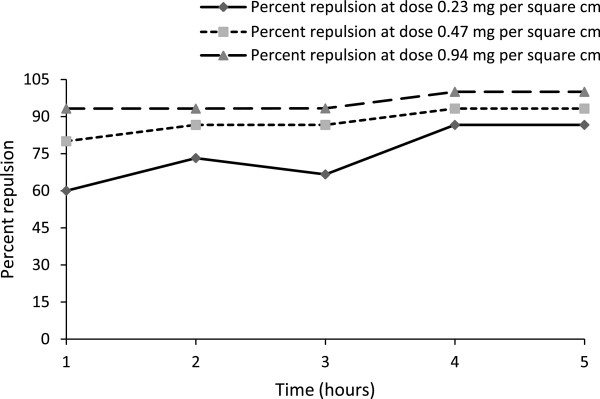
**Repellency records of chloroform fraction per hour interval up to 5 hours.** As dose of chloroform fraction of ethanol extract of rhizome of the plant increased, the repellency property (percent repulsion) of the fraction against the pest also increased. With increasing duration of exposure the repellency property varied (mostly increased), however, within 4 h of exposure duration the repellency property became constant.

## Discussion

A wide range of stored food commodities including grain, flour, peas, beans, nuts, dried fruits and spices were affected by *T. castaneum* [14]. A number of synthetic agents (e.g. methoprene, permethrin, cypermethrin, deltamethrin and fenvalerate etc) were identified for good activity against *T. castaneum*, however, use of these agents has led to problems such as environmental disturbances, increasing costs of application, pest resurgence, pest resistance to pesticides and lethal effects on non-target organisms, in addition to direct toxicity to users [5]. To minimize use of synthetic pesticides and to avoid pollution of the environment, natural pesticide and repellent substances have been searched for pest control during recent times [15]. Plant products having considerable pesticidal potential are gaining tremendous importance in recent years because such products minimize disadvantages associated with synthetic agents [16]. Botanicals used as pesticides presently constitute about 1% of the world pesticide market [17].

Chloroform soluble fraction of *D. quercifolia* have both pesticidal and pest repellency activities against *T. castaneum*. High mortality rate was observed at higher doses (96.60% mortality at 1.77 mg/cm^2^ and 90.00% mortality at 0.88 mg/cm^2^) (Table 2, Figure 1). On the other hand, good pest repellency activity was observed even at lower doses (Table 3, Figure 2). Both pesticidal and pest repellency activities of chloroform soluble fraction of *D. quercifolia* are helpful in controlling pest (*T. castaneum*) of our stored food commodities. No pesticidal and pest repellency activities petroleum ether, ethyl acetate and methanol soluble fractions suggesting the compound(s) worked against *T. castaneum* present in chloroform soluble fraction. Although, 3,4-dihydroxybenzoic acid highly active against both gram positive and gram negative bacteria [12], no activity of the compound was found against *T. castaneum*, indicating some other compound(s) of chloroform soluble fraction were responsible for activity against the pest. Hence, further investigations should be done to isolate the pesticidal and pest repellent compound(s) from this chloroform soluble fraction as well as toxicological studies.

## Conclusions

Among petroleum ether, ethyl acetate, chloroform and methanol soluble fractions of ethanol extract of rhizome of *D. quercifolia*, only the chloroform soluble fraction showed significant pesticidal activity against the pest. Furthermore, the fraction also showed significant pest repellency activity against the pest. Isolated compound 3,4-dihydroxybenzoic acid was inactive against the pest. Overall, it can be stated that good pesticidal and pest repellency activities of the rhizome of *D. quercifolia* suggesting its suitability as botanical pesticide in controlling *T. castaneum* of stored food commodities.

## Methods

### Plant materials

The fresh rhizomes of *D. quercifolia* J. Smith was collected in the month of October from mango trees of Khamar Bari, Lakshmidurpara, Lakshmipur, Bangladesh (year). The plant was taxonomically identified by Professor A. T. M. Naderuzzaman, Department of Botany, University of Rajshahi, Rajshahi, Bangladesh and its voucher specimen (No. 1939) had been deposited.

The rhizomes were first washed with water to remove adhering dirt, cut into small pieces, sun dried for three days and finally dried at 45°C for 36 h in an electrical oven [18]. After complete drying, the entire portions were pulverized into a coarse powder with the help of a grinding machine (FFc-15, China) and were stored in an air tight container for further use [19].

### Extraction of plant materials

The powder materials (600 g) were extracted with ethanol (3 L) in a Soxhlet apparatus (Quickfit, England). The extraction was continued for 72 h at 65°C. The extract was filtered through filter paper. The filtrate was concentrated under reduced pressure at 50°C in a rotary vacuum evaporator to afford a blackish green mass (52.4 g). The blackish green mass was further extracted with petroleum ether, chloroform, ethyl acetate and methanol, and dried under reduce pressure to afford petroleum ether (7.5 g), chloroform (7.8 g), ethyl acetate (5.5 g) and methanol (8.4 g) fractions, respectively [20].

### Isolation of 3, 4-dihydroxy benzoic acid

**Figure 3:**
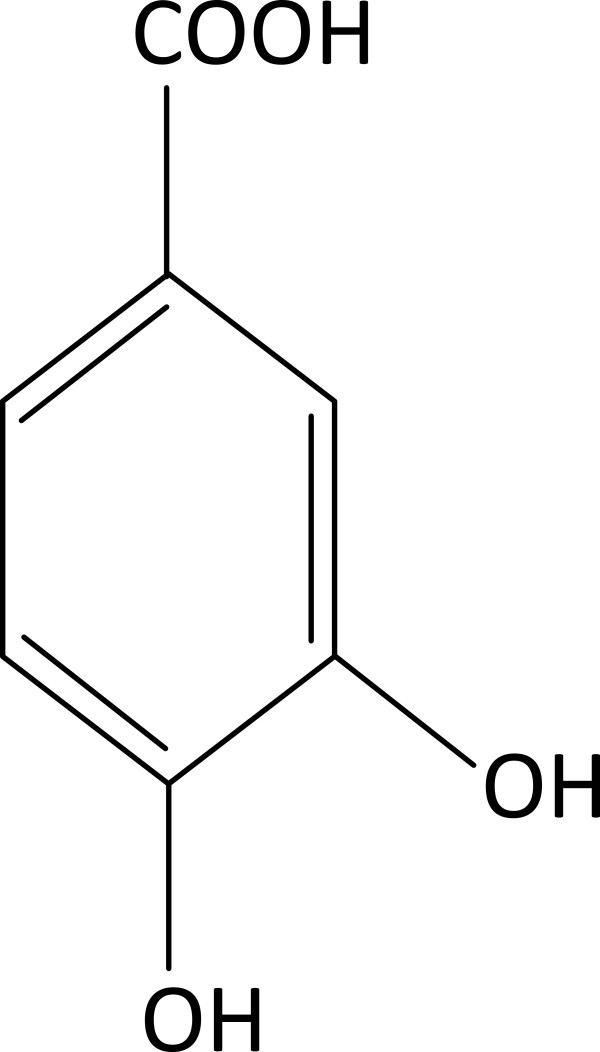
**Structure of compound 1 (3, 4-dihydroxy benzoic acid).** The compound has three functional groups. Two hydroxyl groups at 3 and 4 positions and one carboxyl group at 1 position.

The ethyl acetate soluble fraction was subjected to column chromatography using chloroform and methanol of increasing polarity. Column chromatography yielded 32 fractions. The fractions eluting with 10-25% methanol in chloroform were subjected to preparative TLC (mobile phase 15% methanol in chloroform) to give compound 1 (89 mg). In solubility test, compound 1 was sparingly soluble in water and freely soluble in ethyl acetate, methanol and acetone. The liquid chromatography/electrospray-mass spectroscopy (LC/ES-MS) in the positive ion mode of compound 1 showed molecular [M + H]^+^ peak at *m/z* 154.8 corresponding to a molecular formula of C_7_H_6_O_4_. The IR spectrum exhibited bands at 1240, 1375, 1739, 2877, 2908 and 2985 cm^-1^. The ^1^H-NMR, ^13^C-NMR, HSQC and HMBC spectral data of compound 1 was in good agreement with spectral data of 3,4-dihydroxybenzoic acid (Figure 3) published in literature [21].

### Collection and maintenance of pest

The Tribolium species, *T. castaneum* (Herbst) used in the present experiment was originally received from the Crop Protection Department of the University of Newcastle, U.K. and were reared in the Crop Protection and Toxicology Laboratory, Department of Zoology, University of Rajshahi, Bangladesh.

*T. castaneum* were maintained in 1 L glass jar containing food medium. A filter paper was placed inside each jar for easy movement of the pest. The jar was covered with a filter paper at the top and kept in an incubator at 30 ± 0.5°C.

A standard mixture of wheat flour and powdered brewers yeast in the ratio of 19: 1 was used as food medium to culture the pest. Both flour and yeast were previously passed through a 250 micrometer aperture sieve and mixed thoroughly using an electric blender. The food was sterilized in an oven at 120°C for 6 h. Food was not used until at least 15 d after sterilization to allow its moisture content to equilibrate with that of environment.

### Screening for pesticidal activity

Screening for pesticidal activity was carried out using surface film method [22–24], is a simple and widely used method. The working solution was prepared by dissolving 100 mg experimental sample in 2 ml mixed solvent (50% chloroform + 50% methanol) in a vial. For each sample similar three vials was prepared.

Thirteen clean and dried petridishes (size of each is 60 mm, area of each is 28.26 cm^2^) were taken for each sample. Four petridishes were marked by 50, 25, 12.5 and 6.25 mg. One ml working solution (prepared previously) was poured into the 50 mg petridish and agitated clockwise, anticlockwise, left to right and right to left to further confirm the uniform dispersion. One ml solvent (50% chloroform + 50% methanol) was added to that vial from which 1 ml had been used and mixed uniformly. From this vial, 1 ml solution was poured into the 25 mg petridish and agitated similarly for uniform dispersion. Using this serial dilution technique, likewise sample was poured into 12.5 mg and 6.25 mg petridishes and agitated similarly for uniform dispersion. The above processes were continued two times further using two remaining vials of working solution and eight remaining petridishes. Then the layers of dispersed sample into the petridishes were air dried. One ml solvent (50% chloroform + 50% methanol) was poured and dispersed into control petridish and air dried.

The pests were collected by sieving and ten pests were applied on each layer of dispersed sample into the petridish. This process is continued for each petridish. Then the number of pests have died were recorded after passing 24 and 48 h.

### Pest Repellency Test

Pest repellency test was conducted using filter paper disc method [22, 24, 25]. The working solution was prepared by dissolving 60 mg experimental sample in 2 ml mixed solvent (50% chloroform + 50% methanol) in a vial. For each sample similar three vials was prepared.

Nine clean and dried petridishes (size of each is 90 mm) and Nine filter papers (size-90 mm) were taken for each sample. Three petridishes were marked by 30, 15 and 7.5 mg. Three filter papers were taken for these three petridishes and each filter paper was cut (by scissors) into equal two parts through centre where one part can be used as control part and other part can be used as treated part. For 30 mg petridish with its filter paper, treated part of filter paper was taken at outer background of the petridish and one ml working solution (prepared previously) was dispersed uniformly thorough out this part of filter paper and air dried. Then this part of filter paper was joined with its control part using transparent adhesive tape and placed into the 30 mg petridish using forceps. For 15 mg petridish with its filter paper, treated part of filter paper was taken at outer background of 15 mg petridish. One ml solvent (50% chloroform + 50% methanol) was added to that vial from which 1 ml had been used and mixed uniformly. From this vial, 1 ml solution was dispersed uniformly throughout the treated part of filter paper and air dried. Then this part of filter paper was joined with its control part using transparent adhesive tape and placed into the 15 mg petridish using forcep. Similar works was done for 7.5 mg petridish with its filter paper. The above processes were continued two times further using two remaining vials of working solution and six remaining petridishes and filter papers.

The pests were collected by sieving and ten pests were applied on the filter paper at the center of the petridish. This process was continued for each petridish. Then the number of pests have repelled were counted per hour interval up to 5 h. The percentages of repellency were determined and results were provided through ANOVA after transforming them into arcsin percentage value.

### Statistical analysis

The percent mortality was subjected to statistical probit analysis [26] and the dose-mortality relationship was expressed as a median lethal dose (LD_50_). The repellency values in the recorded data were calculated for percent repellency, which was again transformed by arcsine transformation for the calculation of analysis of variances (ANOVA). Means values were compared using ANOVA (two factors without replication) (Additional File 1: Supplementary Table 1).

## Electronic supplementary material

Additional file 1: Table S1: ANOVA (two factor without replication) for repellency record data through Arcsin transformation. (DOCX 17 KB)
