# Supplementary material for: Pesticidal and pest repellency activities of rhizomes of Drynaria quercifolia (J. Smith) against Tribolium castaneum (Herbst)
Source: Biol Res. 2014 Oct 1;47(1):51. doi: 10.1186/0717-6287-47-51 (PMC4195996; doi:10.1186/0717-6287-47-51)
Supplement: Supplementary file 1 — Additional file 1: Table S1: ANOVA (two factor without replication) for repellency record data through Arcsin transformation. (DOCX 17 KB) [file 40659_2014_50_MOESM1_ESM.docx]

**Additional file 1: Table S1 ANOVA (two factor without replication) for repellency record data through Arcsin transformation.**

| **Source of Variations** | **SS** | **df** | **MS** | **F-ratio** | **P-value** |
| --- | --- | --- | --- | --- | --- |
| Between dose levels | 1073.86 | 2 | 536.93 | 83.23 | 4.42E-06 |
| Between time intervals | 575.46 | 4 | 143.86 | 22.30 | 0.000214 |
| Error | 51.60 | 8 | 6.45 |  |  |
| Total | 1700.94 | 14 |  |  |  |
